# Association Between Pain Self-Efficacy and Adherence to Hemodialysis Regimen

**DOI:** 10.3390/jcm15124824

**Published:** 2026-06-21

**Authors:** Ioanna Mitsia, Vasiliki Matziou, Maria Polikandrioti, Sofia Zyga, Victoria Alikari

**Affiliations:** 1 Post Graduate Program “Applied Nursing Science-Nursing of Renal Diseases”, Department of Nursing, National and Kapodistrian University of Athens, 10561 Athens, Greece; ioannamitsia@gmail.com (I.M.);; 2 Department of Nursing, University of West Attica, 12243 Athens, Greece; 3 Department of Nursing, University of Peloponnese, 22100 Tripolis, Greece

**Keywords:** pain self-efficacy, adherence, hemodialysis

## Abstract

**Background/Objectives**: Pain is a common symptom in patients undergoing hemodialysis (HD) and may influence their quality of life. Pain self-efficacy may play an important role in self-management and adherence behaviors. This study aimed to examine the association between pain self-efficacy and adherence to the HD regimen in patients undergoing HD. **Methods**: In this descriptive and cross-sectional study, 199 patients undergoing HD from a single private hospital (convenience sample) in Athens, Greece, completed the Greek-Simplified Adherence Questionnaire-HD (GR-SMAQ-HD) to assess adherence and the Pain Self-efficacy Questionnaire (PSEQ) to assess pain self-efficacy. Sociodemographic and clinical data were also recorded. Bivariate analyses and multiple linear regression were performed to identify factors associated with adherence. Statistical significance was set at *p* < 0.05. **Results**: Patients demonstrated moderate levels of pain self-efficacy (mean PSEQ = 33.96 ± 9.74) and moderate adherence to the HD regimen (mean GR-SMAQ-HD = 4.78 ± 2.54). No significant correlation was found between pain self-efficacy and adherence in bivariate analysis (rho = 0.125, *p* = 0.221). However, in multivariate analysis, pain self-efficacy was a significant independent predictor of adherence (β = 0.056, *p* = 0.032). Longer duration of End-Stage Renal Disease (ESRD) (β = −0.158, *p* < 0.001), higher pill burden (rho = −0.237, *p* = 0.030) were associated with lower adherence. Marital status was also a significant predictor of adherence (β = 1.631, *p* = 0.016). The model explained 24% of the variance in adherence (Adjusted R^2^ = 0.24). **Conclusions**: Pain self-efficacy may indirectly affect adherence to the HD regimen, although its direct effect is modest. Adherence appears to be negatively influenced by pill burden and ESRD duration, while social support may play an important role.

## 1. Introduction

Chronic kidney disease is a major public health problem that significantly impacts the functionality and quality of life of patients. Patients may progress to End-Stage Renal Disease (ESRD) requiring renal replacement therapy (hemodialysis or peritoneal dialysis) [1].

Despite significant advances in hemodialysis (HD) technology, patients undergoing HD still face serious symptoms in daily life. One of the most common and burdensome symptoms is pain [2]. It varies in both intensity and anatomic location, with musculoskeletal pain or pain related to vascular access being frequently reported. The causes of pain in this population are various and include comorbidities, complications of ESRD, as well as factors related to the HD process itself. In addition, the treatment process may contribute to pain through venipuncture, muscle cramps, or prolonged immobility during the HD session. Despite its frequency and severity, pain in patients undergoing HD remains insufficiently studied and often under-recognized, although its intensity has been compared to the pain experienced by patients in palliative care. These changes, both on a physical and psychological level, lead to social distress, anxiety, and depressive symptoms [3].

The experience of pain concerns not only the physical dimension of an individual’s health but also psychological factors related to how the individual perceives and manages pain. In this context, the concept of pain self-efficacy is of great interest. Pain self-efficacy refers to an individual’s belief that they can function and manage their life despite the presence of pain. The concept is based on Albert Bandura’s theory, which introduced the term self-efficacy [4]. According to this theory, belief in one’s abilities influences health behavior and performance, and the decision to adhere to the regimen. Previous studies [5,6] have shown that high pain self-efficacy is associated with the use of coping strategies (relaxation, movement, activity adaptation), better functioning and adjustment, and less fear of movement. Thus, self-efficacy can predict treatment adherence, health-related attitudes, physical activity, and patients’ ability to effectively self-manage their condition. On the contrary, low pain self-efficacy is associated with increased fear, avoidance of activities, and greater perceived pain intensity, as well as a tendency to reject the treatment regimen [7].

In this context, patients’ perceptions of pain may play a crucial role in health behaviors. This psychological dimension may also influence patients’ ability to adhere to the HD regimen. Adherence to the HD regimen refers to the extent to which patients follow treatment instructions for medication intake, dietary and fluid restrictions, and attendance at scheduled HD sessions. The frequency of non-adherence to the diet and fluid restriction is approximately 60% [7,8]. Studies show that patients exhibit selective non-adherence to medications, particularly to phosphorus binders, because they must be taken with meals at least 3 times daily [9]. Poor adherence has been associated with increased morbidity, more frequent hospitalizations, and worse clinical outcomes. Specifically, non-adherence to fluid restriction and HD sessions may lead to respiratory distress and acute pulmonary edema, whereas non-adherence to dietary restrictions, particularly regarding foods rich in potassium, sodium, and phosphorus, may lead to cardiac arrhythmias and renal osteodystrophy [10].

Although pain self-efficacy has been extensively studied in various chronic diseases, such as musculoskeletal disorders [11] or cancer [12], the relationship between pain self-efficacy and adherence to the HD regimen in patients undergoing HD remains insufficiently explored. Understanding this relationship may contribute to the design of targeted interventions that will enhance patients’ active participation in their treatment and improve both their clinical and psychosocial outcomes. Therefore, this study aims to investigate the levels of pain self-efficacy and adherence to the HD regimen among patients undergoing HD. Also, the association between pain self-efficacy and adherence to the HD regimen was studied, as well as between adherence and sociodemographic and clinical characteristics. Based on the previous literature [5,6,7], the following hypotheses were formulated: (H1) pain self-efficacy is significantly associated with adherence to the HD regimen, and (H2) pain self-efficacy is a significant predictor of adherence to the HD regimen.

## 2. Materials and Methods

This is a descriptive and cross-sectional study conducted in a single private hospital in Athens (the capital city of Greece). A convenience sample was used based on feasibility and accessibility. The inclusion criteria were: patients undergoing HD for at least 6 months, ability to read and sign the informed consent form, and age > 18 years. The exclusion criteria were: patients with severe physical problems (rheumatoid arthritis, cognitive impairment or dementia, acute neurological disorders), visual and hearing impairment, or psychiatric problems (psychotic disorders, severe depressive disorder, bipolar disorder), that may compromise completion of the questionnaires. Information concerning patients’ cognitive, psychiatric, and eye/hearing status was obtained from medical records. All patients were invited to participate. Of the total 222 patients, 205 met the eligibility criteria, and finally, 199 completed the questionnaires. No formal sample size calculation was performed due to convenience sampling. Data were collected in September 2023.

To collect data, participants completed two questionnaires:

The GR-SMAQ-HD was used to measure adherence to the HD regimen [13]. The scale consists of a total of eight items categorized into three dimensions of adherence to HD regimen: (a) Medication Adherence (items 1–4, e.g., “Do you always take your medication at the appropriate time?”; (b) Attendance at HD sessions (items 5 and 6, e.g., “Last month, on average, how many minutes was the session cut off on your own initiative?”; and the remaining assess adherence to Dietary/Fluid Restrictions (items 7 and 8, e.g., “During the last week, how often did you follow the instructions on fluid restrictions?”. Items 1–3 are dichotomous (Yes/No), while the remaining items are answered on a 5-point Likert-type scale. The total score ranges from 0 to 8, with higher scores indicating greater adherence to the HD regimen. The Cronbach’s Alpha coefficient for the entire scale in the Greek population is 0.742 [13]. The scale has been used in several studies [14,15,16].

The Pain Self-Efficacy Questionnaire (PSEQ), developed in 1999 by Nicholas [17], evaluates patients’ confidence in performing usual activities and maintaining normal personal and social functioning despite chronic pain. The questionnaire consists of 10 self-reported items rated on a 7-point Likert-type scale (0 = not at all confident to 6 = completely confident). The total score is calculated by summing item responses and ranges from 0 to 60. Higher scores indicate greater perceived pain self-efficacy. No cut-off score exists. The scale requires approximately 5 min to complete. In this study, the Greek version of the scale was used (Cronbach’s alpha for the Greek population 0.98) [18]. The scale has been widely used in various populations, including patients with chronic musculoskeletal pain [19] and low back pain [11].

In addition, sociodemographic (gender, professional status, marital status) and clinical characteristics (time after ESRD diagnosis, HD vintage, vascular access, and comorbidities) of the patients were recorded.

Data analysis was performed using SPSS version 28 (IBM Corp., Armonk, NY, USA). The level of statistical significance was set at *p* < 0.05. Initially, descriptive statistical analysis was performed. Quantitative variables are presented as mean and standard deviation (SD) or as medians and interquartile range (IQR). The normality of quantitative variables was assessed using the Kolmogorov–Smirnov or Shapiro–Wilk test. Qualitative variables are presented as absolute (N) or relative (%) frequencies.

Subsequently, bivariate analyses were conducted. Specifically, to compare the scores of the adherence and pain self-efficacy scales with the demographic and clinical characteristics, the following tests were used: (a) the Mann–Whitney U test for comparing a non-normally distributed quantitative variable with a dichotomous variable, (b) the Kruskal–Wallis test for comparing a non-normally distributed quantitative variable with a categorical variable with at least three categories. To control for Type I error due to multiple comparisons, the Bonferroni correction was applied, setting the level of statistical significance at 0.05/k (k = number of comparisons). Spearman’s correlation coefficient was used to analyze the relationship between two quantitative variables when at least one variable did not follow a normal distribution. There was no missing data.

Multiple linear regression analysis was performed using the stepwise method to identify independent factors associated with adherence to the HD regimen. Regression assumptions, including linearity, normality, homoscedasticity, and absence of multicollinearity, were checked and met. Variables that were significant in the bivariate analyses or clinically relevant variables were included in the model. The GR-SMAQ-HD score is a bounded scale (0–8); however, it was treated as a continuous variable for regression analysis, as is common in similar studies [19]. Results are presented as regression coefficients (β), 95% confidence intervals (95% CI), and *p*-values.

The research protocol was approved by the Ethics Committee of the Department of Nursing of the National and Kapodistrian University of Athens (apr. number 386, 8 July 2022), as well as by the Scientific Board of the hospital where the study was conducted (22 November 2022). The study was conducted in accordance with the ethical principles of the Declaration of Helsinki and complied with the General Data Protection Regulation 2016/679 (GDPR).

All participants were informed orally and received written information about the purpose of the study, and written consent was obtained before participation. Patients’ participation was voluntary, and complete confidentiality was maintained regarding patients’ information. The security of the relevant material was preserved. The participants’ anonymity was guaranteed, and the results obtained were used exclusively for this research.

## 3. Results

### 3.1. The Sample

The study sample consisted of 199 patients with ESRD undergoing hemodialysis. Of these, 119 (59.70%) were male. The mean age was 60.2 (SD ± 12.1). The most common comorbidities among the patients were diabetes mellitus (42.21%) and hypertension (50.75%). The median time after diagnosis of the ESRD was 6.00 (4.00–9.00) years, while the median HD vintage was 48 (36–60) months. The median number of pills per day was 7.00 (5.00–8.00). Finally, the majority of patients underwent HD via arteriovenous fistula (AVF). Table 1 presents the sociodemographic and clinical characteristics of the sample.

### 3.2. Descriptive Data of the Scales

The mean PSEQ score was 33.96 (SD = 9.74), indicating that patients demonstrated moderate pain self-efficacy. In the present study, the Cronbach’s Alpha coefficient for the PSEQ scale was 0.89, and for the GR-SMAQ-HD, it was 0.76. Regarding patients’ adherence to the HD regimen, the mean GR-SMAQ-HD score was 4.78 (SD = 2.54), indicating moderate adherence (Table 2).

### 3.3. Correlation Between Pain Self-Efficacy and Adherence

Correlation between the Gr-SMAQ-HD and the Pain Self-Efficacy Questionnaire: No statistically significant correlation was found between patients’ adherence to HD regimen and pain self-efficacy (rho = 0.125, *p* = 0.221) (Table 3).

### 3.4. Bivariate Analysis

Association between the GR-SMAQ-HD and patients’ sociodemographic and clinical data: Patients’ adherence differed statistically according to marital status (*p* < 0.001), with married patients having higher adherence compared to unmarried and widowed/divorced patients. Also, patients who did not live alone had statistically significantly higher adherence than those who lived alone (*p* = 0.03) (Table 4).

The Mann–Whitney U test was used for dichotomous variables; Kruskal–Wallis test was used for categorical variables with more than two categories. For variables with more than two categories, significant Kruskal–Wallis tests were followed by post hoc pairwise comparisons with Bonferroni adjustment.

Table 5 presents the results of the association between the GR-SMAQ-HD score and clinical characteristics of the sample. The adherence score was negatively correlated with time after diagnosis of ESRD (rho = −0.439, *p* < 0.001), HD vintage (rho = −0.345, *p* < 0.001), and number of pills per day (rho = −0.237, *p* = 0.030). No significant correlation was observed between the duration of HD sessions and adherence (rho = −0.062, *p* = 0.545). Adherence differed significantly regarding the vascular access. Specifically, patients with arteriovenous fistula had higher adherence than those with arteriovenous graft (*p* = 0.008). Adherence did not differ significantly regarding the mode of transportation to the HD unit (Kruskal–Wallis H = 1.042, *p* = 0.791).

Kruskal–Wallis test was used for categorical variables; Spearman’s correlation for continuous variables. For significant Kruskal–Wallis tests, post hoc pairwise comparisons were performed with Bonferroni adjustment.

### 3.5. Multiple Linear Regression Analysis with Patient Adherence to the HD Regimen as the Dependent Variable

A multiple linear regression analysis was performed to identify independent predictors of adherence to the HD regimen (GR-SMAQ-HD score). Variables that were significantly associated with adherence in univariate analyses, as well as variables with clinical relevance or theoretical importance, were included. Although no significant correlation was observed between PSEQ and adherence in the bivariate analysis, PSEQ emerged as a significant predictor in the multivariate model. According to the results, the PSEQ score was a significant predictor of adherence, as for each one-point increase in the PSEQ score, the GR-SMAQ-HD score is expected to increase by 0.056 units (*p* = 0.032). Marital status was also a significant predictor of adherence, as married individuals are expected to have a higher GR-SMAQ-HD score than unmarried individuals by 1.631 units (*p* = 0.016). In addition, for each additional year after diagnosis of ESRD, the GR-SMAQ-HD score is expected to decrease by 0.158 units (*p* < 0.001). The overall regression model was significant (F (4, 194) = 8.47, *p* < 0.001) and explained 24% of the variance (Adjusted R^2^ = 0.24) (Table 6).

## 4. Discussion

The present study examined the levels of pain self-efficacy and adherence to the HD regimen among patients undergoing HD. Furthermore, the association between pain self-efficacy and adherence was tested, highlighting findings that partly align and differ from the international literature.

Patients’ pain self-efficacy was assessed using the Pain Self-Efficacy Questionnaire (PSEQ). Patients demonstrated moderate levels of pain self-efficacy. The results are consistent with previous studies [20] in patients with chronic diseases and especially those with intractable chronic pain, where prolonged exposure to symptoms seems to limit patients’ confidence to manage daily activities effectively. Similar findings have also been reported among patients undergoing HD in the study by Zyga et al. [21], where the mean PSEQ score was 32.5.

One of the findings of the present study was the moderate level of patients’ adherence to the HD regimen. This observation is in line with the international literature [22,23], which shows that non-adherence to dietary and fluid restrictions remains high, reaching up to 60% among participants. Previous studies using the same measurement tool have reported varying levels of adherence. For example, Alikari et al. [13] reported a mean GR-SMAQ-HD score of 6.35. However, in another study [24], the majority of patients demonstrated high medication adherence but lower adherence to diet and fluid restrictions. Factors such as the complexity of the HD regimen, the duration of treatment, the adherence tool used, and psychosocial determinants appear to significantly affect adherence behaviors.

Another important finding of the present study was that there was no correlation between pain self-efficacy and adherence to the HD regimen in bivariate analysis. Although a weak positive correlation was revealed (rho = 0.125), it did not reach statistical significance (*p* = 0.221). Therefore, the research hypothesis (H1) was not supported by the study findings. Our finding differs from previous studies, such as Perdana and Yen [23], which found that patients with higher self-efficacy also had greater adherence to HD treatment.

Although self-efficacy is considered a key factor in the self-management of chronic diseases, the findings, particularly among patients undergoing HD, are not entirely consistent. Saguban et al. [25] found that despite high levels of patients’ self-efficacy, a large proportion of patients under HD were non-adherent to the HD regimen. The researchers emphasize that depression is a major factor that actually contributes to low self-efficacy.

The findings of the present study reveal a more complex relationship. Although no direct association was observed in the bivariate analysis, pain self-efficacy emerged as an independent predictor of adherence in the multivariate analysis, confirming the research hypothesis (H2). For each one-unit increase in the PSEQ score, the GR-SMAQ-HD score is expected to increase statistically significantly by 0.056 units. Although statistically significant, the effect size was small (β = 0.056), suggesting that pain self-efficacy plays a modest role in adherence when other factors are considered. This result is consistent with Bandura’s self-efficacy theory [4] and with evidence [26] that individuals’ beliefs about their abilities influence health-related behavior, especially when multiple confounding factors are considered. The disparity between the two analyses suggests that the effect of pain self-efficacy is not direct but probably indirect or mediated by other variables, which may help explain why no significant association was observed in the bivariate analysis.

Although the multivariate model was statistically significant, it explained only 24% of the variance in adherence, suggesting that a substantial portion of the variability remains unexplained by the variables examined. This reinforces the view that adherence is a multifactorial phenomenon influenced by additional psychological, social, and environmental factors [27] not included in the present study.

Among the clinical variables examined, the time after the diagnosis of ESRD emerged as one of the strongest determinants of adherence to the HD regimen. In particular, a moderate-to-strong negative correlation (rho = −0.439) was observed between adherence scores and time after ESRD diagnosis. This finding indicates that the long time after the diagnosis of ESRD is one of the major factors of low adherence. Therefore, long-term experience with the disease is associated with progressively lower adherence levels. The negative correlation between disease duration and adherence is consistent with findings from other studies [28], highlighting the phenomenon of “treatment fatigue,” according to which long-term exposure to chronic illness may lead to behavioral fatigue and coping exhaustion.

In contrast, a moderate but statistically significant negative correlation (rho = −0.345) was observed between adherence score and HD vintage. This means that the longer a patient has been on HD, the lower the level of adherence to the HD regimen. Patients undergoing long-term hemodialysis often show reduced interest or exhaustion in adhering to restrictions, leading to lower adherence [29].

Also, the association between adherence score and number of pills per day was weaker but statistically significant (rho= −0.237), indicating that increased pill burden is associated with lower adherence. Similar findings have been reported in previous studies [30,31], which demonstrated that a higher number of prescribed medications is linked to reduced adherence. This may be explained by the complexity of the treatment regimen and patients’ beliefs about their treatment. Patients often consider the treatment ineffective or experience difficulties due to variable side effects (e.g., gastrointestinal disorders caused by phosphate binders) [32]. This result highlights the importance of simplifying the HD treatment regimen to improve adherence.

Also, in our study, patients with AVF had higher adherence than those with AVG. The observed association between adherence and the type of vascular access is also consistent with some studies [33], in which the use of an AVF is associated with improved clinical outcomes and possibly greater patient involvement in health care. In contrast, catheter use has been associated with increased complications and lower quality of life, factors that may negatively affect adherence [34]. It should be noted that in our study, the number of patients with AVG was small (n = 13), which may have limited the reliability of group comparisons.

Regarding the effect of demographic factors on patients’ adherence to the HD regimen, our study found that marital status was a significant predictor of increased adherence. More specifically, married patients are expected to have significantly higher adherence than single patients by 1.631 points. The significant effect of marital status on adherence is supported by previous findings [35], which indicate that married patients or those with a strong support network demonstrate higher adherence to treatment instructions. In our study, although having children was not significantly correlated with adherence (*p* = 0.06), a trend towards higher adherence was observed among patients with children. This finding may be related to an increased sense of responsibility or a supportive family environment. In addition, patients who were not living alone had statistically significantly higher adherence scores than those living alone, further highlighting the importance of social and family support. This observation is consistent with previous studies [36], indicating that a supportive environment facilitates adherence by providing practical assistance and emotional support in managing chronic illness.

No statistically significant association was observed between adherence and gender, educational level, occupational status, HD session duration, or mode of transportation to the HD unit. These sociodemographic and clinical factors do not appear to play a prominent role in adherence to the HD regimen.

An important finding of the study is that the predominant type of vascular access in the sample was the AVF. At this point, it should be emphasized that the repeated cannulation of the AVF is a major source of pain in patients undergoing HD. This type of pain can affect physical functioning and emotional well-being, mainly due to the frequent and long-term repeatability of the procedure. Continuous exposure to painful invasive procedures can lead to increased perceived burden of treatment and decreased willingness to adhere to the HD regimen [37].

The present study has some limitations. The cross-sectional nature of the study does not allow the inference of causal relationships between variables. The fact that the study is single-center limits the generalizability of the results. Additionally, the questionnaires were distributed during the HD session, where potential distractions may have influenced patients’ responses. Also, confounders such as pain severity and psychological status may influence both PSEQ and GR-SMAQ-HD scores. Adherence and self-efficacy were assessed through questionnaires and not through objective measurements (biochemical markers, interdialytic weight gain), which may be influenced by social desirability bias or patients’ subjective assessment. Also, the small number of patients in some subgroups (e.g., AVG) may limit the statistical power and reliability of the comparisons. Finally, the low percentage of explained variance suggests that potentially important factors (e.g., depression, anxiety, health literacy, relationship with health professionals) were not included.

The findings of the study highlight the need for a systematic assessment of patients’ beliefs about their ability to manage pain. The adoption of nurse-led educational interventions that enhance self-efficacy, such as promoting self-management and self-care through increased disease-related knowledge and a patient-centered approach, particularly among patients with long-term disease, may contribute to improved adherence to the HD regimen. For patients with limited social support, nurses should include family members and caregivers in educational programs. Within an interdisciplinary framework, care should integrate medical and pharmacological approaches with psychosocial support, including physicians, specialist nurses, psychologists, and social workers.

## 5. Conclusions

Although the results of our study did not reveal a direct relationship between pain self-efficacy and adherence to the HD regimen, it emerged that pain self-efficacy may contribute indirectly to adherence, probably due to the multifactorial nature of adherence behavior. In addition, a longer time after diagnosis of ESRD and a higher pill burden seem to negatively affect adherence, which is possibly related to therapeutic exhaustion and reduced coping capacity. Also, patients with a supportive environment, including those who are married or have children, demonstrated higher levels of adherence, indicating the important role of social and family support.

## Figures and Tables

**Table 1:** Sociodemographic and clinical characteristics of participants (Ν = 199).

	N	(%)
Gender		
Males	119	59.70
Females	80	40.20
Marital status		
Unmarried	32	16.08
Married	120	60.30
Divorced or Widowed	47	23.61
Having children		
Yes	111	55.77
No	88	44.22
Living alone		
Yes	57	28.64
No	142	71.35
Educational level		
Primary School	51	25.62
Secondary School	35	17.58
High school	75	37.68
University	38	19.09
Occupational status		
Unemployed	12	6.03
Household	22	11.05
Self-employed	18	9.04
Private employee	36	18.09
Public employee	4	2.01
Retired	107	53.76
Age	Mean: 60.2 (SD ^1^ ± 12.11)	
Comorbidities *		
Diabetes Mellitus	84	42.21
Hypertension	101	50.75
Glomerulonephritis	20	10.05
Polycystic Kidney Disease	28	14.07
Other	6	3.01
Previous Peritoneal Dialysis		
Yes	4	2.01
No	195	97.98
Mode of transportation to the HD Unit		
Private Car	116	58.29
Ambulance	21	10.55
Taxi	35	17.58
On foot	27	13.56
Vascular access		
AVF ^3^	135	67.83
CVC ^4^	51	25.62
AVG ^5^	13	6.53
	Median (IQR) ^2^	
Time after diagnosis of ESRD ^6^	6.00 (4.0–9.0)	
HD vintage (months)	48 (36–60)	
HD Session duration (hours)	4.00 (3.50–4.0)	
Number of pills per day	7 (5–8)	

* Participants could have more than one comorbidity; therefore, percentages do not sum to 100%. ^1^ SD: Standard Deviation. ^2^ Interquartile Range. ^3^ AVF: Arteriovenous Fistula. ^4^ CVC: Central Venous Catheter. ^5^ AVG: Arteriovenous Graft. ^6^ ESRD: End-Stage Renal Disease.

**Table 2:** Descriptive characteristics of the scales.

	Mean (SD) ^1^	Median (IQR) ^2^	Min–Max	Cronbach’s Alpha
PSEQ	33.96 (±9.74)	33.50 (27.00–40.00)	12.00–57.00	0.88
GR-SMAQ-HD	4.78 (±2.54)	5.00 (3.00–7.00)	0.00–8.00	0.77

^1^ SD: Standard Deviation. ^2^ Interquartile Range.

**Table 3:** Correlation between adherence and pain self-efficacy.

		GR-SMAQ-HD
PSEQ	rho	0.125
	*p*-value	0.221

Statistical significance: *p* < 0.05.

**Table 4:** Association of sociodemographic characteristics with adherence.

Sociodemographic Characteristics	Mean ± SD ^1^	Statistic Test (*p*-Value)
Gender		Mann–Whitney U = 1092.0 (*p* = 0.620)
Males	4.88 ± 2.55	
Females	4.65 ± 2.55	
Marital status		Kruskal–Wallis H = 16.101, df = 2, (*p* < 0.001)
Unmarried	3.88 ± 2.28	
Married	5.60 ± 2.41	
Widowed or Divorced	3.42 ± 2.32	
Children		Mann–Whitney U = 669.0 (*p* = 0.06)
No	4.24 ± 2.43	
Yes	5.94 ± 2.58	
Living alone		Mann–Whitney U = 585.0 (*p* = 0.03)
Yes	3.56 ± 2.38	
No	5.21 ± 2.47	
Educational level		Kruskal–Wallis H = 3.082, df = 3, (*p* = 0.544)
Primary school	4.87 ± 2.98	
Secondary school	5.06 ± 2.13	
High school	4.89 ± 2.34	
University	3.79 ± 3.15	
Occupational status		Kruskal–Wallis H = 2.131, df = 2, (*p* = 0.345)
Unemployed or Household	5.11 ± 2.35	
Employed	4.76 ± 2.84	
Retired	4.17 ± 2.69	

^1^ SD: Standard Deviation. Statistical significance: *p* < 0.05.

**Table 5:** Association of clinical characteristics with adherence.

	Mean ± SD ^1^	Statistic Test (*p*-Value)
Time after diagnosis of ESRD ^2^ (years)	-	rho = −0.439 (<0.001)
HD vintage	-	rho = −0.345 (<0.001)
HD session duration (hours)	-	rho = −0.062 (0.545)
Number of pills per day	-	rho = −0.237 (0.030)
Mode of transportation to the HD unit		Kruskal-Wallis H = 1.042, df = 3, (*p* = 0.791)
Private Car	4.77 ± 2.58	
Ambulance	4.50 ± 1.90	
Taxi	5.00 ± 2.42	
On foot	3.50 ± 3.51	
Vascular access		Kruskal-Wallis H = 9.630, df = 2 (*p* = 0.008)
Arteriovenous Fistula (AVF)	5.22 ± 2.50	
Central Venous Catheter (CVC)	4.26 ± 2.53	
Arteriovenous Graft (AVG)	2.50 ± 1.85	

^1^ SD: Standard Deviation. ^2^ ESRD: End-Stage Renal Disease. rho = Spearman’s correlation coefficient. Statistical significance: *p* < 0.05.

**Table 6:** Multiple linear regression analysis with adherence as the dependent variable.

Independent Variables	β (95% CI ^1^)	*p*-Value
PSEQ	0.056 (0.005–0.107)	0.032
Marital status		
Unmarried (reference category)		
Married	1.631 (0.309–2.950)	0.016
Widowed/Divorced	0.029 (−1.484–1.543)	0.969
Time after diagnosis of ESRD ^2^ (years)	−0.158 (−0.241–[−0.077])	<0.001

^1^ CI: Confidence Interval. ^2^ ESRD: End-Stage Renal Disease. Model fit: F(4, 194) = 8.47, Statistical significance *p* < 0.001; Adjusted R^2^ = 0.24.

## Data Availability

The data presented in this study are available on request from the corresponding author. The data are not publicly available due to ethical and privacy restrictions.

## Abbreviations

The following abbreviations are used in this manuscript:

AVF	Arteriovenous Fistula
AVG	Arteriovenous Graft
CI	Confidence Interval
CVC	Central Venous Catheter
Df	degrees of freedom
ESRD	End-Stage Renal Disease
GR-SMAQ-HD	Greek-Simplified Adherence Questionnaire in Hemodialysis
HD	Hemodialysis
IQR	Interquartile Range
PSEQ	Pain Self-Efficacy Questionnaire
SD	Standard Deviation
